# Dengue Virus Dysregulates Master Transcription Factors and PI3K/AKT/mTOR Signaling Pathway in Megakaryocytes

**DOI:** 10.3389/fcimb.2021.715208

**Published:** 2021-08-26

**Authors:** Anismrita Lahon, Ravi P. Arya, Akhil C. Banerjea

**Affiliations:** ^1^Laboratory of Virology, National Institute of Immunology, New Delhi, India; ^2^Kusuma School of Biological Sciences, Indian Institute of Technology Delhi, New Delhi, India; ^3^Institute of Advanced Virology, Kerala, India

**Keywords:** dengue virus, thrombopoietin, AKT, mTOR, NF-E2, GATA-2, MEG-01

## Abstract

Dengue virus (DENV) infection can cause either self-limited dengue fever or hemorrhagic complications. Low platelet count is one of the manifestations of dengue fever. Megakaryocytes are the sole producers of platelets. However, the role of both host and viral factors in megakaryocyte development, maturation, and platelet production is largely unknown in DENV infection. PI3K/AKT/mTOR pathway plays a significant role in cell survival, maturation, and megakaryocyte development. We were interested to check whether pathogenic insult can impact this pathway. We observed decreased expression of most of the major key molecules associated with the PI3K/AKT/mTOR pathway in DENV infected MEG-01 cells. In this study, the involvement of PI3K/AKT/mTOR pathway in megakaryocyte development and maturation was confirmed with the use of specific inhibitors in infected MEG-01 cells. Our results showed that direct pharmacologic inhibition of this pathway greatly impacted megakaryopoiesis associated molecule CD61 and some essential transcription factors (GATA-1, GATA-2, and NF-E2). Additionally, we observed apoptosis in megakaryocytes due to DENV infection. Our results may suggest that DENV impairs PI3K/AKT/mTOR axis and molecules involved in the development and maturation of megakaryocytes. It is imperative to investigate the role of these molecules in the context of megakaryopoiesis during DENV infection to better understand the pathways and mechanisms, which in turn might provide insights into the development of antiviral strategies.

## Highlights

•Activation of PI3K/AKT/mTOR pathway is essential for megakaryocyte maturation and development (megakaryopoiesis).
•DENV impairs PI3K/AKT/mTOR axis in megakaryocytes.
•DENV impairs megakaryopoiesis-related key molecules like GATA-1, GATA-2, and NF-E2.
•DENV infection leads to apoptosis in megakaryocytes.

**Graphical Abstract:**
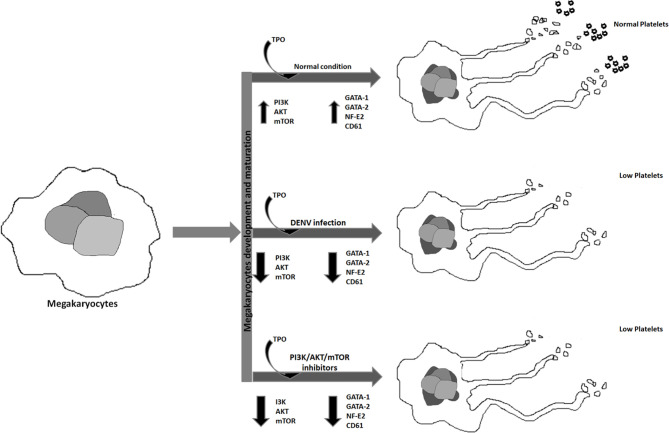
Graphical abstract represents the effect of DENV infection and different inhibitors on megakaryopoiesis.

## Introduction

Platelets are indispensable for maintaining body functions as they are the regulators of hemostasis, thrombosis, and inflammatory responses (Li et al., 2017). These are anucleate cells (2–4 μm in diameter) produced by bone marrow derived giant precursor cells, called megakaryocytes (50–100 µm in diameter) (Szalai et al., 2006; Geddis, 2010; Machlus and Italiano, 2013). Megakaryocytes undergo a series of highly orchestrated processes to produce platelets. Mature megakaryocytes with long cytoplasmic processes produce pro-platelets and these pro-platelets finally convert into platelets, and the whole process is called megakaryopoiesis. Different factors strongly influence maturation and successful production of platelets by megakaryocytes, such as growth-promoting factor thrombopoietin (TPO) and PI3K/AKT/mTOR, transcription factors GATA-1 and GATA-2, STAT molecules, and NE-F2 (Szalai et al., 2006; Geddis, 2010; Machlus and Italiano, 2013). Among these, molecules involved in PI3K/AKT/mTOR signaling are crucial for megakaryopoiesis (Chen et al., 2018). Transcription factors like GATA1, GATA-2, and NF-E2 have been shown as significant contributors to the development of megakaryocytic lineage (Shivdasani et al., 1995). Obstruction in platelet formation by megakaryocytes occurs when any of these factors are affected by pathogen(s) or imbalance in homeostasis.

Megakaryocytes are the target of several viral pathogens. Hantaan virus (HTNV), a *Bunyaviridae* family member, can efficiently replicate in differentiating megakaryocytic cells (Lütteke et al., 2010). Junin virus, an *Arenavirus* family member, can infect megakaryocytes and impairs thrombopoiesis by decreasing proplatelet formation and platelet release (Pozner et al., 2010). The human immunodeficiency virus, human herpes virus, and human cytomegalovirus (Gonelli et al., 2002; Costantini et al., 2006; Chen et al., 2017) are few examples of other viruses capable of infecting megakaryocytes and hampering platelet production.

DENV infection is the most common mosquito-transmitted infection globally, with ~390 million reported cases per year (Murray et al., 2013). Symptomatic DENV infected individuals develop fever, headache, vomiting, muscle and joint pains, and a characteristic skin rash or progress to severity (Kularatne, 2015). Life-threatening dengue hemorrhagic fever or dengue shock syndrome is a severe condition with major symptoms like bleeding, thrombocytopenia and plasma leakage, or low blood pressure (Simmons et al., 2012).

DENV possesses a positive-sense single-stranded RNA genome of ~11 kb, translated into a single polyprotein which further processed into structural proteins (capsid, prM/membrane, and envelope) and non-structural proteins (NS1-NS5) by host or viral proteases enzymes (Bollati et al., 2010). DENV has been classified into four serotypes, DENV1–4, and infection with two or more serotypes can cause severe life-threatening disease (Halstead and Deen, 2002; Holmes and Twiddy, 2003).

The effect of DENV infection in the megakaryocyte is poorly understood. Host factors tightly regulate megakaryopoiesis or platelet production, and impairment of these factors may lead to low platelet production by megakaryocytes. DENV infection in megakaryocytes and different cell types of megakaryocytic lineage (UT-7, MEG-01, K562) is well reported (Basu et al., 2008; Clark et al., 2012; Clark et al., 2016; Vogt et al., 2019). In this study, we focused on host factors that are crucial for megakaryopoiesis during DENV infection by using MEG-01 cells.

## Methods

### DENV Stock Preparation

Virus stocks were prepared by infecting monolayers of C6/36 cells with dengue virus serotype-2 strain (DENV2) NGC. Infected cells were maintained in L-15 medium (Gibco Life Technologies), supplemented with 2% fetal bovine serum (FBS) and antibiotic antimycotic solution (Himedia Laboratories). Virus containing cell supernatants were harvested, filtered, and stored at -70°C till further use. Virus stocks were quantified by qRT-PCR. To attain a sufficient titer, virus concentration was performed using amicon centrifugal filter tubes (Millipore). The concentrated virus was used to infect megakaryocytic cell line MEG-01 (kindly provided by Dr. Sankar Bhattacharyya, THSTI, India). Heat-inactivation of virus stocks was performed at 55°C for 1 h.

### Cell Culture, Treatment, and DENV Infection

MEG-01 cells, a human megakaryoblastic leukemia cell line, were grown in RPMI medium (Lonza) with 10% FBS and antibiotic solution (Himedia Laboratories) with 5% CO_2_. For further differentiation, MEG-01 cells were treated with growth factor TPO (100 ng/mL; PeproTech) for 24 h prior to experiments. For DENV infection, MEG-01 cells were incubated with DENV for 3 h at 37°C, washed with PBS, and resuspended in RPMI-1640 medium with 10% FBS/1X antibiotics and kept at 37°C with 5% CO_2_ in a cell culture incubator for different experiments. To inhibit different cellular mechanisms, AKT, PI3K, PKCα, and mTOR inhibitors (AKT inhibitor IV (5.0 µM), Ly294002 (10.0 µM), and HBBDE (10.0 µM) from Sigma Aldrich; Torin1 from Cell Signaling Technology) were used respectively as per-requisite of experiments after infection.

### Western Blotting and Antibodies

Mock and DENV infected MEG-01 cells were harvested on day 5^th^ post-infection and washed with cold PBS. Cells were lysed with RIPA lysis buffer by vortexing several times and incubated on ice for 30 min. Supernatants from whole lysates were separated by centrifugation at 10,000g for 30 min at +4°C, and protein quantification was done using BCA method (Pierce BCA Protein Assay Kit; Thermo Fisher Scientific). An equal amount of total protein was mixed with sample loading dye and resolved on 8–12% SDS-polyacrylamide gel. The same were transferred on nitrocellulose membrane (# SCNJ8101XXXX101, MDI, Advanced Microdevices Ltd., India). The membrane was blocked with 5% skimmed milk or BSA solution for 1 h at room temperature. Afterward, the membrane was subjected to incubation with primary antibody, washed, and incubated with suitable secondary antibodies (Jackson ImmunoResearch, USA). The membrane was washed with TBST and developed on x-ray film by using chemiluminescence solutions. Following antibodies used in this study were obtained from Cell Signaling Technology [anti GATA-2 (# 4595), anti Akt (pan) (#2920), anti phospho-Akt (Thr308; #4056), anti phospho-Akt (Ser473; #4060) anti mTOR (#2983), anti phospho-mTOR (Ser2448; #2971), anti p70 S6 Kinase (#9202), anti PKCα (#2056), anti Bcl-2 (# 15071)]; Santa Cruz Biotechnology [anti-GAPDH (#Sc-32233)]; and Sigma Aldrich [anti NF-E2 (#HPA001914) and anti-DENV NS1 (# SAB2702308)].

### Quantitative Real-Time PCR for Viral Replication

Viral load in the supernatants was estimated by extracting viral RNA from culture supernatants and harvested from DENV infected MEG-01 cells using QIAamp Viral RNA Mini Kit (Qiagen). Eluted RNA samples were mixed using TaqMan fast virus 1-step master mix (Applied Biosystems) with DENV-2 specific primers and probe and subjected to qRT-PCR. Viral load was estimated applying the standard curve method using DENV-2 transcripts (Lahon et al., 2016).

### Apoptosis Assay

MEG-01 cells (1X10^5^) were infected with 1 MOI of DENV or left alone. Cells were harvested at a given time point and washed with cold PBS. Cells were subjected to staining with annexin-V (#556420, BD Biosciences) and propidium iodide (#556547, BD Biosciences) and incubated at room temperature in the dark for 20 min according to manufacturer instructions. Stained cells were washed and resuspended in PBS, and data were acquired on BD canto flow cytometry instrument. Data were analyzed using BD FACS-Diva software (BD Biosciences).

### Flow Cytometry Analysis of CD61 and DENV Envelope Protein

Mock and DENV infected MEG-01 cells, with or without treatment of different inhibitors of PI3K, AKT, and mTOR pathway, were washed and stained for CD61 (PE-CD61, # 555754 BD Bioscience) and washed with PBS and 2% FBS. Cells were fixed with paraformaldehyde and analyzed on a flow cytometry analyzer (BD-Bioscience). For staining of DENV envelope protein in MEG-01 cells, infected and control cells were fixed with IC-fixation buffer (Thermofisher Scientific) in the dark and washed twice with permeabilization buffer. Then, cells were incubated with 4G2 antibody (# MAB10216, EMD-Millipore) for 30 min and washed with permeabilization buffer followed by incubation with anti-mouse FITC conjugated secondary antibody (Thermofisher Scientific). Cells were washed and analyzed on a flow cytometry analyzer (BD-Bioscience).

### Statistical Analysis

All the cell culture experiments were done in triplicates, and results were represented as mean ± standard error. All graphs were prepared in Graphpad Prism, and statistical analysis was performed by non-parametric Student’s t-test and p < 0.05 considered as significant. ImageJ software was used to analyze Western blot images, and GAPDH was used as endogenous control to calculate fold changes.

## Results

### DENV Infection Impaired Expression of PI3K and AKT in MEG-01 Cells

PI3K and AKT pathway activation has been shown very crucial to regulate platelet production by megakaryocytes (Kaushansky, 2005; Di Buduo et al., 2016). Disruption of the said pathway in megakaryocytes may impede the production of platelets (Nakao et al., 2008). PI3K/AKT pathway is essential for cell survival, inhibition of apoptosis, development, and maturation of megakaryocytes. Flaviviruses have been shown to modulate the same pathway for their survival (Mannová and Beretta, 2005). However, how DENV modulates PI3K/AKT signaling in megakaryocytes is poorly understood. To understand the underlying mechanism, we evaluated the expression of AKT and associated proteins involved in PI3K/AKT pathway in MEG-01 cells during DENV infection.

**Figure 1:**
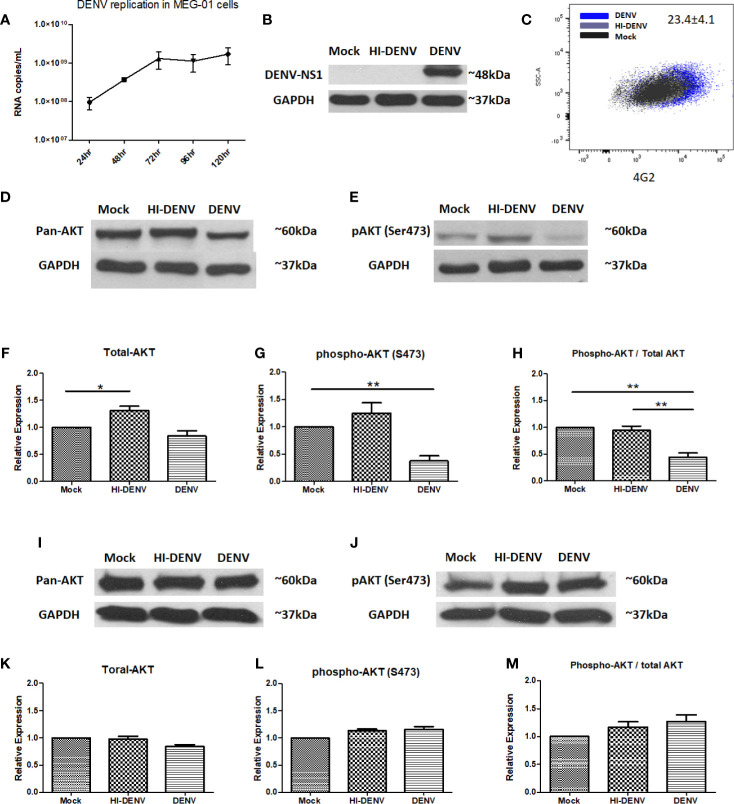
DENV infects MEG-01 cells and modulates the expression of AKT. **(A)** MEG-01 cells were infected with 1 MOI of DENV, and replication was determined from cell culture supernatants collected at day 1 post-infection through day 5 post-infection by using RT-PCR. **(B)** Replication was further confirmed by immunoblotting for DENV NS1 (~48 kDa) in 5^th^-day post infected cell lysate and **(C)** flow cytometric detection of DENV envelope protein using 4G2 antibody staining of infected cells (mean ± SE of infected cells = 23.4 ± 4.1). **(D, E)** Immunoblotting of pan-AKT and phosphorylated AKT (Ser473) in mock-infected, DENV inactivated virus treated, and DENV infected MEG-01 cell lysate at 5^th^ day post-infection. **(F–H)** Relative expression of pan-AKT and phospho-AKT was calculated by using the densitometry method and presented as column graphs. The ratio of phospho-AKT and pan-AKT is also presented as column graphs. **(I, J)** Immunoblot of pan-AKT and phospho-AKT from the lysate of mock, inactivated virus treated, and DENV infected HEK293 cells and **(K–M)** presentation of relative expression of pan-AKT and phospho-AKT as column graphs of mock control, inactivated virus treated, and DENV infected HEK293 cells. (*pValue < 0.05; **pValue < 0.01).

TPO treated MEG-01 cells were incubated with the DENV-2 virus (1 MOI). Virus replication was determined from cell culture supernatants collected at given time points by using RT-PCR (**Figure 1A**). Replication was further confirmed by immunoblotting for DENV non-structural protein 1 (NS1, an expected band of ~48 kDa was detected) in 5^th^ -day post infected cell lysate (**Figure 1B**) and flow cytometric detection of DENV envelope protein using 4G2 antibody staining of infected cells (mean ± SE of infected cells = 23.4 ± 4.1; **Figure 1C**). However, heat-inactivated virus failed to express NS1 and viral envelope protein (**Figures 1B, C** **)**. We checked the expression of pan AKT and phospho-AKT with or without infection. DENV infected MEG-01 cells showed deteriorated expression of pan-AKT and phosphorylated AKT (ser473) levels at 5^th^ day post infection when compared with mock cells (**Figures 1D–H**, p < 0.05). The ratio of phospho-AKT and total AKT was found significantly reduced in infected cells (**Figure 1H**, p < 0.05). To confirm the role of virus replication, we utilized heat-inactivated (HI) DENV stock. We found higher levels of pan AKT in the cells with inactivated virus compared with control or virus infected MEG-01 cells (**Figures 1D** **–H**; p < 0.05). We also analyzed the expression of phospho-AKT in the lysate of mock and DENV infected cells at 3, 4, and 5 days post infection (**Supplementary Figure S1**). Additionally, we utilized HEK-293 cells, a different cell lineage from MEG-01 cells, to understand the effect of DENV replication in two different cell types. We observed a slight decrease in the pan-AKT expression in HEK-293 cells with DENV infection than controls, albeit we could not find any difference in the expression of phospho-AKT (ser473) (**Figures 1I** **–M**; p < 0.05).

Activated PI3K components regulate AKT and its downstream signaling. PI3K has three important components: a regulatory subunit p85 and catalytic subunit isoforms p-110α and p110β. The p85 binds to the N-terminus of the p110 subunit *via* its iSH2 domain and regulates its activity (Backer, 2010). So, our next logical question was whether DENV also influences the upstream molecule PI3K.

**Figure 2:**
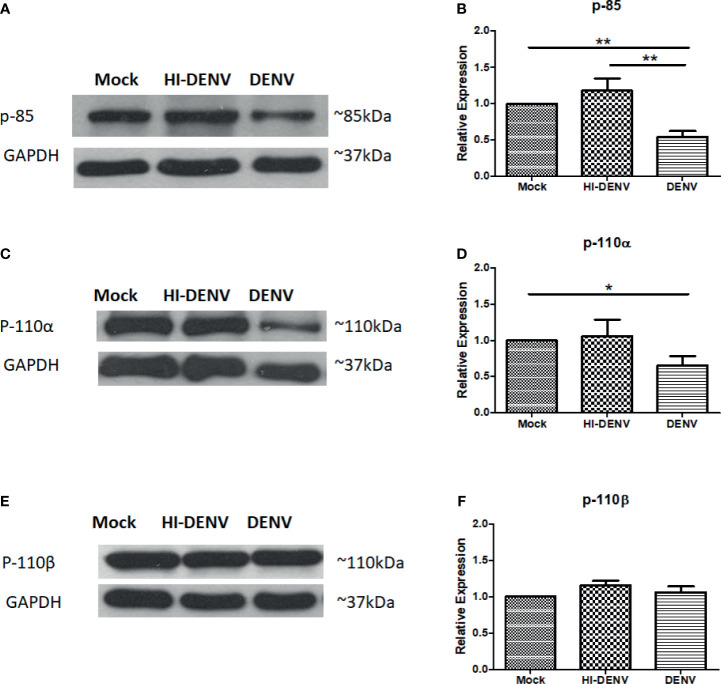
Immunoblotting of PI3K regulatory and catalytic unit proteins. Representative immunoblots and relative expression of **(A, B)** p-85, **(C, D)** p110-α, and **(E, F)** p-110-β in mock-infected, DENV inactivated virus treated, and DENV infected MEG-01 cell lysate. (*pValue < 0.05; **pValue < 0.01).

The expression of p85 and one of the catalytic subunit, p110α, was observed low in DENV infected MEG-01 cells than in uninfected controls (**Figures 2A**–**D**; p < 0.05). No change was observed in the levels of p110β in DENV infected cells (**Figures 2E, F**). However, with inactivated virus, increased expression of p110β was noted (**Figures 2E** **, F**; p < 0.05). Our results suggest that DENV may have profound effects on PI3K/AKT pathway in infected megakaryocytes.

### Effect of DENV on mTOR and Associated Molecules in Megakaryocytes

**Figure 3:**
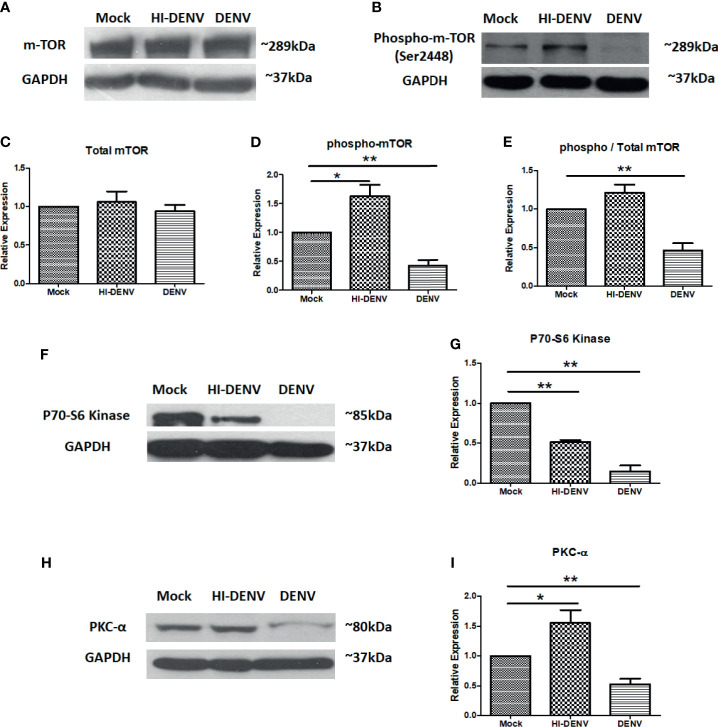
Immunoblotting of mTOR and associated proteins. **(A, B)** Representative immunoblots of total mTOR and phospho-mTOR (Ser2448) in mock, DENV inactivated virus treated, and DENV infected MEG-01 cell lysate. Graphical representation of the expression of mTOR **(C)** total mTOR, **(D)** phospho-mTOR, and **(E)** ratio of phospho and total mTOR expression. Representative immunoblots and relative expression of **(F, G)** P70-S6 kinase and **(H, I)** PKC-α in mock-infected, DENV inactivated virus treated, and DENV infected MEG-01 cell lysate. (*pValue < 0.05; **pValue < 0.01).

Activation of mTOR, a downstream molecule of PI3K/AKT pathway, is also required for megakaryocyte development and platelet production (Liu et al., 2011; Elagib et al., 2018). Next, we checked the expression of mTOR and associated molecules in the conditions we opted in previous experimentation. We observed that DENV replication in MEG-01 cells reduced the expression of phosphorylated mTOR than control cells (**Figures 3A** **–E**; p < 0.05), and no change was observed in total mTOR expression, while inactivated virus enhanced the expression of p-mTOR (**Figures 3A**–**E**; p < 0.05). The ratio of total mTOR and p-mTOR showed reduced activation of mTOR during infection (**Figure 3E**; p < 0.01). mTOR and its associated signaling molecules formed two different complexes, mTORC1 and mTORC2. These complexes activate other signaling molecules in their downstream pathways. mTORC1 and mTORC2 complexes activate P70-S6 Kinase and PKCα, respectively. These molecules had been screened to confirm which mTOR complex was affected during DENV infection in MEG-01 cells. We observed that both P70S6 Kinase (mTORC1) and PKC-alpha (mTORC2) were downregulated with DENV infection (**Figures 3F**–**I**; p < 0.05). However, the expression of PKC-alpha (mTORC2) was observed more than P70-S6 Kinase (mTORC1), suggesting that the mTORC2 complex is less affected than mTORC1 in megakaryocytes. With inactivated virus, we observed higher expression of PKC-alpha (mTORC2). However, P70-S6 Kinase (mTORC1) was still at lower levels than control cells (**Figures 3F**–**I**; p < 0.05).

### Expression of Pro and Anti-Apoptotic Markers in DENV Infected Megakaryocytes

**Figure 4:**
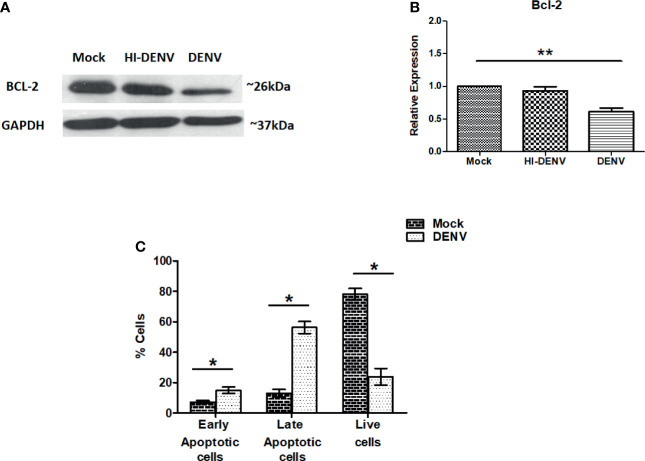
DENV infection leads to apoptosis in MEG-01 cells. **(A, B)** Immunoblotting of Bcl-2 protein in mock-infected, DENV inactivated virus treated, and DENV infected MEG-01 cell lysate and represented as column graphs. **(C)** DENV infected and control MEG-01 cells were stained with FITC conjugated Annexin-V and Propidium Iodide and analyzed on flow cytometer. Graph representation of early apoptosis (only Annexin-V positive cells), late apoptosis (Annexin-V + PI positive cells), and live cells (double negative cells). (*pValue < 0.05; **pValue < 0.01).

So far, we have observed that DENV infection is impairing the PI3K/AKT/mTOR axis in MEG-01 cells, which may activate molecules responsible for cell death/apoptosis. Bcl-2 is an anti-apoptotic protein that keeps other apoptotic proteins in inactive form and inhibits apoptosis (Senichkin et al., 2020; Kehr and Vogler, 2021). Immunoblotting of Bcl-2 was carried out on previously explained conditions. A higher level of Bcl-2 was noted in the cells treated with inactivated virus, and low expression was observed in DENV infected cells (**Figures 4A** **, B**; p < 0.05). The immunoblot result may suggest that DENV infection drives MEG-01 cells towards apoptosis. To further confirm, apart from immunoblotting, we utilized annexin-V and PI staining to understand apoptosis in MEG-01 cells. On day 5^th^ post-infection, cells were washed and subjected to analysis on flow cytometry for apoptosis. Our flow cytometry results revealed that DENV infection significantly (p = 0.001) increased early apoptotic and late apoptotic cells than controls on the 5^th^ post day infection (**Figure 4C**).

### DENV Reduces the Factors Responsible for Megakaryocyte Development and Maturation

**Figure 5:**
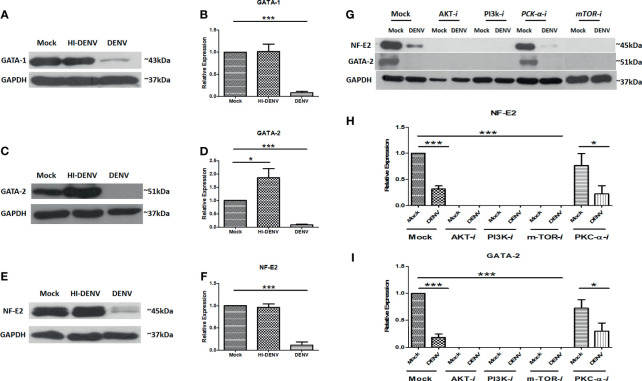
Immunoblotting of development and maturation-specific transcription factors in MEG-01 cells. Immunoblot and graphical representation of the expression of GATA-1 **(A, B)**, GATA-2 **(C, D)**, and NF-E2 **(E, F)** in mock, DENV inactivated virus treated, and DENV infected MEG-01 cell lysate. Effect of different inhibitors of AKT (AKT-IV), PI3K (Ly294002), mTOR (Torin-1), and PKCα (HBBDE) on the expression of most affected transcription factors **(G)** and their graphical presentation **(H, I)** during DENV infection. (*pValue < 0.05; ***pvalue < 0.001).

To confirm our hypothesis that DENV compromised the megakaryopoiesis and lower the production of platelets, we checked the transcription factors and surface markers involved in development and maturation of megakaryocytes. NF-E2, GATA-1, and GATA-2 are common transcription factors involved in the megakaryocyte maturation and development and taking the megakaryocytes towards megakaryopoiesis and platelet production (Shivdasani et al., 1995; Szalai et al., 2006; Geddis, 2010; Machlus and Italiano, 2013; Chen et al., 2018). We utilized similar experimental conditions and observed that DENV significantly reduced the expression of GATA-1, GATA-2, and NF-E2 (**Figures 5A** **–F**; p < 0.05). However, inactivated virus significantly increased the expression of GATA-2 (**Figures 5C** **, D**; p < 0.05) and had no effect on NF-E2 and GATA-1.

### Role of PI3K/AKT/mTOR Pathway in Megakaryocyte Development and Maturation

Further, we wanted to confirm the role of PI3K/AKT/mTOR signaling in the megakaryopoiesis. So, we utilized inhibitors of PI3K, AKT, mTOR, and PKC-α and observed the levels of transcription factors NF-E2 and GATA-2 by Western blot. We found that inhibitors of AKT, mTOR, and PI3K completely abolished the expression of NF-E2 and GATA2 with or without infection, suggesting that these proteins had a significant role as regulatory molecules of megakaryopoiesis process. However, PKC-α inhibitor showed a reduction in the expression of NF-E2 and GATA-2 only with DENV infection, suggesting the role of PKC-α in the derailing of megakaryopoiesis process during infection (**Figures 5G** **–I**; p < 0.05).

**Figure 6:**
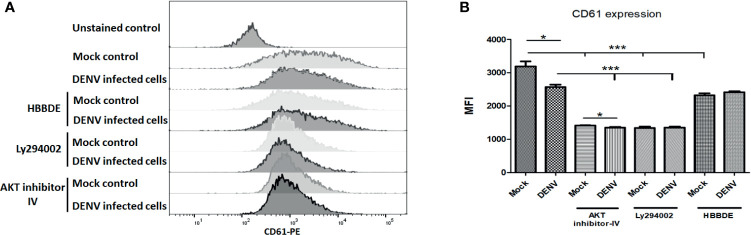
Flow cytometry of CD61 in mock and DENV infected MEG-01 cells. MEG-01 cells were mock-treated or treated with inhibitors of AKT (AKT-IV), PI3K (Ly294002), and PKCα (HBBDE). **(A)** Histograms from different conditions represent the expression of CD61 and **(B)** column graph presentation of median fluorescence intensity (MFI) of CD61 in cells with the above-stated condition. (*pValue < 0.05; ***pvalue < 0.001).

CD61 is a glyco-protein present on the surface of megakaryocytes and platelets. During megakaryopoiesis, the expression of CD61 increases on megakaryocytes. Given the importance of CD61 in megakaryopoiesis and platelet production, we also observed expression of CD61 with or without above-mentioned inhibitors and found that except PKC-α inhibitor AKT and PI3K reduced CD61 expression significantly (p < 0.01; **Figures 6A, B**) observed by flow cytometry. DENV infection significantly reduced the expression of CD61 in MEG-01 cells (p < 0.05). However, only with AKT inhibitor, DENV infection was observed with further reduction in CD61 expression (p < 0.05). No significant difference was observed between control and DENV infected cells treated with PI3K or PKC-α inhibitor. These results confirmed the involvement of PI3K/AKT pathway in the megakaryopoiesis, which is abolished by DENV infection in megakaryocytes.

## Discussion

The low platelet count is a severe condition in DENV infection which may lead to dengue shock syndrome or dengue haemorrhagic fever that leaves the patient at a significant risk of spontaneous bleeding. Although there is a wealth of literature on DENV, the mechanism(s) leading to such condition have been a long-standing question and not explored enough regarding which host or viral factor(s) is/are crucially involved. Therefore, to find the processes in which viral infection leads to low platelet counts, we aimed to study the effect of DENV infection in megakaryocytes.

Megakaryocyte development is a multifactorial and complex process and depends upon TPO stimulation. PI3K/AKT axis is crucial for megakaryocyte survival and has been reported for maturation, development, and platelet production (Kaushansky, 2005; Nakao et al., 2008; Di Buduo et al., 2016). TPO treatment to primary megakaryocytes and megakaryocytic cell line regulates expression of p27^Kip1^, a cyclin dependent kinase inhibitor through PI3K/AKT pathway, and drives them towards platelet production (Nakao et al., 2008). We have observed impaired expression of pan-AKT and phospho-AKT (Ser473) in MEG-01 cells infected with DENV, whereas the same has no effect on the phosphorylation status of AKT in HEK-293 cells, a different cell lineage. Thus, DENV does not have a similar effect on cells from different lineages. Additionally, inactivated virus showed an increase in the expression of pan-AKT and phospho-AKT (Ser473), suggesting the reduction of AKT expression is due to active replication of DENV. On the contrary, Japanese Encephalitis Virus and DENV have been shown to enhance the phosphorylation of AKT in a mouse neuroblastoma cell line (N18). However, reduction in both pan-AKT and phospho-AKT has been shown by Tick-borne flavivirus infection in HEK-293 cells (Kirsch et al., 2020).

PI3K, an upstream signalling molecule of AKT, and AKT forms a key signaling nexus along with mTOR, which regulates cell survival, metabolism, and differentiation (Jean and Kiger, 2014). This signaling nexus is also required for megakaryopoiesis and platelet production (Kaushansky, 2005; Nakao et al., 2008; Backer, 2010; Liu et al., 2011; Di Buduo et al., 2016). Loss of AKT expression during DENV infection made us to investigate the expression of PI3K and mTOR. PI3K has been made up of different proteins, p-85 (regulatory unit) and p-110α and p110β (catalytic unit). DENV infection in MEG-01 cells was observed with reduced expression of regulatory unit p85 and catalytic unit p110α that suggests that DENV may impair PI3K activity in megakaryocytes. Modulation of PI3K activity by regulating p85 expression has been reported in case of influenza virus (Shin et al., 2007). HCV, another flavivirus, has been shown to modulate PI3K activity in Huh-7 cells and positively regulates HCV translation through Sterol regulatory element-binding proteins (Shi et al., 2016). However, inactivated virus showed upregulation of p110β along with p85 expression in MEG-01, which is less active than p110α (Utermark et al., 2012).

mTOR has been shown to play a key role in megakaryocyte terminal differentiation and megakaryopoiesis human primary megakaryocytes derived from precursor and megakaryocytic cell line (MO7e) (Raslova et al., 2006; Fuhler et al., 2009). mTOR pathway has also been reported to regulate the ploidy nature of cells and size of megakaryocytes by modulating the expression of different downstream effector molecules such as p21 and cyclin D3 and control megakaryocyte differentiation (Raslova et al., 2006). mTOR forms two different complexes, i.e., mTORC1 and mTORC2, by interacting with associate proteins, raptor (mTORC-1) and rictor (mTORC2). Impairment of these molecules in megakaryocytes decreases the normal megakaryopoiesis, leading to low platelet production (Raslova et al., 2006; Fuhler et al., 2009). Different viruses such as Influenza A virus, HCV, or classical Swine Fever Virus have been reported for the modulation of mTORC1 and mTORC2 (Stöhr et al., 2016; Kuss-Duerkop et al., 2017; Luo et al., 2018). During DENV infection, inhibition of mTOR activity is required to induce lipophagy in Hep G2 cells and autophagy in HUVECs (Jordan and Randall, 2017; Kong et al., 2020). DENV infection in megakaryocytes showed a decrease in the phospho-mTOR and P70-S6 Kinase and PKC-α (effector molecules of downstream of mTORC1 and mTORC2). Thus, DENV infection impaired both mTORC1 and mTORC2 pathways and may impair megakaryopoiesis.

We have observed DENV replication enhanced early and late apoptotic cells, suggesting DENV infection induces apoptosis in megakaryocytes. DENV has been shown to inhibit megakaryocytic colony formation and induce apoptosis in TPO-induced megakaryocytes generated from cord blood CD34^+^ cells *in vitro* (Basu et al., 2008). Apoptosis is strictly regulated in several cells and required activation or inhibition of several proteins involved in programmed cell death. Bcl-2 acts as an anti-apoptotic protein by inhibiting other pro-apoptotic proteins (Senichkin et al., 2020; Kehr and Vogler, 2021). DENV infection in MEG-01 cells showed a reduction of Bcl-2 that also supports our finding of apoptosis in megakaryocytes. Human herpes virus-7 infection has been reported to a drastic increase of apoptosis in megakaryocytic cells (Gonelli et al., 2002). Human cytomegalovirus can also induce apoptosis of infected megakaryocytes through the mitochondria-mediated intrinsic pathway (Chen et al., 2017).

Developmental processes of megakaryocytes involved several factors, such as different transcription factors and maturation surface markers. GATA family proteins are involved in this process, i.e., GATA-1 and GATA-2. These proteins are required to regulate polyploidization, cell cycle progression, and expression of megakaryopoiesis specific genes such as GPIIb, PF4, GPIbα, β-TG, GPIX, or GPV through its downstream effector STAT1 (Huang et al., 2009). NF-E2 expression is required for megakaryocyte development and maturation and acts as a crucial molecule that determines the stages of megakaryopoiesis (Levin et al., 1999; Tijssen and Ghevaert, 2013; Noetzli et al., 2019). DENV infection abolished the expression of GATA-1, GATA-2, and NF-E2 proteins, which further confirms the earlier finding of impairment of megakaryopoiesis. Additionally, use of pharmacological inhibitors of PI3K/AKT/mTOR pathway provides the connection between this pathway and megakaryocytes’ maturation (GATA-2 and NF-E2 expression). PI3K, AKT, and mTOR inhibitors have profound effect on the expression of GATA-2 and NF-E2. However, PKCα inhibitor (HBBDE) had lesser effect on the levels of GATA-2 or NF-E2. We also evaluated the expression of another maturation marker, CD61, which present on the surface of megakaryocytes and found reduced in expression during DENV infection (Arya et al., 2021). CD61 is a type-I integral transmembrane glycoprotein that belongs to the integrin family and plays critical role in megakaryocyte development/maturation, platelet activation, and platelet aggregation (Pérez de la Lastra et al., 1997). Application of inhibitors of PI3K/AKT/PKCα molecules suggests a possible correlation between CD61 expression (Levin et al., 1999; Kehr and Vogler, 2021) and PI3K/AKT/PKCα pathways, which was impaired during DENV infection. Thus, our results suggest that DENV infection in megakaryocytes impaired PI3K/AKT/mTOR axis along with maturation molecules (GATA-1/GATA-2/NF-E2/CD61) and leads to apoptosis. Although our work highlights interesting molecular mechanisms associated with DENV pathogenesis in megakaryocytes, limitations to the current study could not be ignored due to unavailability of *in vivo* models or megakaryocytes isolated from DENV infected patients.

## Conclusions

In general, PI3K/AKT/mTOR axis is necessary for normal cell growth and survival. Additionally, this pathway is also required for megakaryopoiesis. Our results suggest that DENV infection altered the PI3K/AKT/mTOR pathway, which further impaired the development, maturation, and terminal differentiation of megakaryocytes and enhanced apoptosis. Pharmacological inhibitors also confirmed the requirement of these molecules in megakaryopoiesis. This study could be one of the small steps towards finding the complex biological process of megakaryopoiesis affected by DENV replication.

## Data Availability Statement

The raw data supporting the conclusions of this article will be made available by the authors, without undue reservation.

## Author Contributions

Conceptualization: AL and AB. Experiment design: AL and RA. Writing—original draft: AL. Writing—review and editing: AL, RA, and AB. Methodology and investigation: AL and RA. Data analysis: AL and RA. Laboratory resources: AB. Funding acquisition: AL. All authors contributed to the article and approved the submitted version.

## Funding

This study was supported by Department of Science and Technology (DST), Government of India, New Delhi, India in the form of research grant to AL. AL is a recipient of DST‐INSPIRE Faculty award from DST, GOI, New Delhi.

## Conflict of Interest

The authors declare that the research was conducted in the absence of any commercial or financial relationships that could be construed as a potential conflict of interest.

## Publisher’s Note

All claims expressed in this article are solely those of the authors and do not necessarily represent those of their affiliated organizations, or those of the publisher, the editors and the reviewers. Any product that may be evaluated in this article, or claim that may be made by its manufacturer, is not guaranteed or endorsed by the publisher.

## Supplementary Material

Supplementary Figure 1 Immuno-blot of phospho-AKT (S473) in mock infected and DENV infected MEG-01 cells at 3, 4, and 5 days post infection.
