# Supplementary figures and images for: Dengue Virus Dysregulates Master Transcription Factors and PI3K/AKT/mTOR Signaling Pathway in Megakaryocytes
Source: Front Cell Infect Microbiol. 2021 Aug 26;11:715208. doi: 10.3389/fcimb.2021.715208 (PMC8427595; doi:10.3389/fcimb.2021.715208)

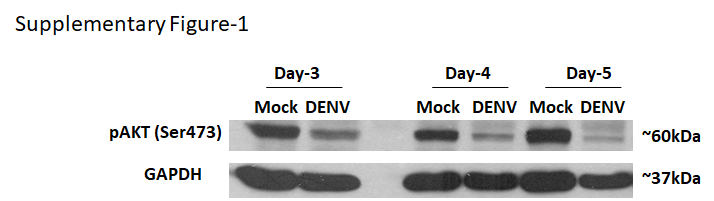

Supplement: Supplementary Figure 1 — Immuno-blot of phospho-AKT (S473) in mock infected and DENV infected MEG-01 cells at 3, 4, and 5 days post infection. [file Image_1.tif]
